# The Association between IgG and Disease Severity Parameters in CF Patients

**DOI:** 10.3390/jcm10153316

**Published:** 2021-07-28

**Authors:** Michal Gur, Yael Ben-David, Moneera Hanna, Anat Ilivitzki, Adi Weichhendler, Ronen Bar-Yoseph, Yazeed Toukan, Kamal Masarweh, Lea Bentur

**Affiliations:** 1 Pediatric Pulmonary Institute and CF Center, Rappaport Children’s Hospital, Rambam Health Care Campus, Haifa 3109601, Israel; m_gur@rambam.health.gov.il (M.G.); Y_BENDAVID@rambam.health.gov.il (Y.B.-D.); m_hanna@rambam.health.gov.il (M.H.); adiw2035@gmail.com (A.W.); r_bar-yoseph@rambam.health.gov.il (R.B.-Y.); y_toukan@rambam.health.gov.il (Y.T.); M_KAMAL@rambam.health.gov.il (K.M.); 2 Rappaport Faculty of Medicine, Technion—Israel Institute of Technology, Efron St 1, Haifa 3525422, Israel; a_ilivitzki@rambam.health.gov.il; 3 Pediatric Radiology Unit, Ruth Rappaport Children’s Hospital, Rambam Health Care Campus, Haifa 3109601, Israel

**Keywords:** cystic fibrosis, immunoglobulin G, lung clearance index, disease severity, computed tomography

## Abstract

Assessing disease severity in patients with cystic fibrosis (CF) is essential when directing therapies. Serum immunoglobulin G (IgG) levels increase with disease severity. Lung clearance index (LCI) is recognized as an outcome measure for CF clinical trials. Our aim was to evaluate the correlations between IgG and disease severity markers. This was a single-center retrospective study, evaluating association between IgG and markers of severity in CF patients (including clinical characteristics, lung spirometry, LCI, clinical scores and computed tomography (CT) scores) during stable conditions. There were 69 patients, age 20.5 ± 11.6 years. Nineteen (27.5%) patients had elevated IgG. IgG correlated positively with LCI (r = 0.342, *p* = 0.005). IgG was higher in pancreatic insufficient (PI) and patients with liver disease (1504.3 ± 625.5 vs. 1229 ± 276.1 mg/dL in PI vs. PS, *p* = 0.023, and 1702.6 ± 720.3 vs. 1256.2 ± 345.5 mg/dL with vs. without liver disease, *p* = 0.001, respectively). IgG also correlated positively with CRP, CT score, and days with antibiotics in the previous year (r = 0.38, *p* = 0.003; r = 0.435, *p* = 0.001; and r = 0.361, *p* = 0.002, respectively), and negatively with FEV1% and SK score (r = −0.527, *p* < 0.001 and r = −0.613, *p* < 0.001, respectively). IgG correlated with clinical parameters, pulmonary functions, and imaging. However, this is still an auxiliary test, complementing other tests, including lung function and imaging tests. Larger multi-center longitudinal studies are warranted.

## 1. Introduction

Cystic fibrosis (CF) arises from mutations in the *CF transmembrane conductance regulator* (CFTR) gene, which is responsible for anion transport across CF airway epithelial cells. Absence of functional CFTR leads to abnormal mucus viscosity and impaired muco-ciliary clearance, resulting in persistent airway colonization with pathogenic bacteria, especially *Pseudomonas aeruginosa* (PA) [1].

Chronic infection and inflammation play a crucial role in developing CF lung disease, and together cause progressive lung damage. However, airway inflammation may be excessive and sustained relative to the infection. There is evidence to suggest that CFTR is a pro-inflammatory gene, contributing to immune dysregulation and, hence, both infection and inflammation are considered hallmarks of CF [1]. In response to pathogens, there are increased amounts of neutrophils in the lungs and elevated various sputum and systemic inflammatory mediators; TNF-α, interleukin (IL)-6, neutrophil elastase, antiprotease and immunoglobulin G (IgG) are increased [2], while anti-inflammatory cytokines, such as IL-10 [3,4] are decreased.

Human IgG and subclasses (IgG1–IgG4) have a prominent role in defending against a variety of microorganisms and trigger effector functions, through binding to cellular Fc-receptors for IgG. They may induce pro-inflammatory responses by recruiting innate immune cells or activating the complement pathway [5]. The association between IgG levels and disease severity in CF was reported in the early 1960s. Immunoglobulin levels were found to increase with age, as well as during pulmonary exacerbations [6]. An association between chronic colonization with PA and elevated IgG levels and its subtypes was found in several studies [3,6,7,8].

Lung clearance index (LCI) is a marker that provides a global measurement of ventilation inhomogeneity LCI has been found to be superior to FEV1 in monitoring early lung disease [9,10], and was recognized as an outcome measure for CF clinical trials [11,12].

Although previous studies examined the association between IgG levels, clinical variables and FEV1 in CF, we are not aware of studies evaluating the association between IgG levels and LCI or low irradiation CT. Thus, our aim was to evaluate the association between IgG levels and markers of disease severity, primarily LCI. Other markers of disease severity included clinical characteristics, lung functions, imaging, and inflammatory markers. Our hypothesis was that IgG would correlate with relatively new markers of disease severity. Such correlations may offer an additional assessment and follow-up tool, where these measurements are not easily available, delineating high-risk patients who need closer surveillance.

## 2. Materials and Methods

This was a retrospective single-center study and was approved by the institutional review board. The study population comprised patients with CF who were followed at our tertiary center between January 2014 and February 2020. The routine follow-up of our patients included spirometry and sputum culture at every routine clinic visit (every three months); blood tests twice a year; LCI once a year; and computed tomography (CT) scans every three years.

Of 80 CF patients followed in our center, 69 had sufficient data to be included in the study. LCI results were available for 66 patients, as three patients could not perform technically acceptable MBW measurements. We used the LCI and spirometry values that were performed at the closest visit to the CT scans, while in a stable clinical state (study visit). Patients who performed neither LCI nor CT scans were not included in the study.

The data retrieved from the patients’ records included:

- Demographics: age, gender
- Weight, height, BMI (at the date of study visit)
- Disease characteristics: pancreatic insufficiency/sufficiency (PI/PS); F508del mutation; CF related diabetes (CFRD); CF liver disease; gastrostomy status; allergic bronchopulmonary aspergillosis (ABPA)
- Sputum cultures: chronic colonization with PA
- Inflammatory markers: IgG and c-reactive protein (CRP) (at the study visit or within three months). Values of IgG were analyzed according to age reference values [13]
- Spirometry results—FVC, FEV1 and FEF 25-75. Reference values were retrieved from Polgar and Quanjer [14]
- LCI—retrieved from multiple breath washout (MBW) measurements, Easy-One Pro, MBW Module (NDD Medical Technologies, Zurich, Switzerland). LCI is defined by the number of functional residual capacity (FRC) turnovers required to washout the nitrogen; thus, elevated LCI (>7) reflects inhomogeneous ventilation [9,15]
- Days of antibiotic treatment for pulmonary exacerbations in the year preceding the study visit (oral (PO), intravenous (IV) and total). Chronic antibiotic therapy, such as azithromycin or treatment for atypical mycobacterium, was not considered to be antibiotic treatment
- Standardized severity score was calculated for each patient at the study visit by the modified Shwachman–Kulczycki (SK). The modified score includes three categories: nutritional status, physical findings, and general activity. Each category is given 5 to 25 points, giving a maximum score of 75 points. A higher score indicates better health [16]
- CT scans were anonymized and a modified Bhalla score was performed by a pediatric radiologist. The score includes the extent of bronchiectasis and number of segments involved, peri-bronchial thickening, mucus plugs, sacculation, bullae, collapse, consolidation, and emphysema. The total score ranges from 0 to 25, with a higher score indicating more severe changes [17].

### Statistical Methods

#### Statistical Analysis Was Performed Using SPSS Version 25

The primary outcome was the correlation between IgG and LCI. The secondary outcomes were the correlations between IgG and the other parameters. Descriptive statistics were used for the demographic variables. The results are expressed as mean ± SD, median (IQR—interquartile range) and % predicted, as applicable. For the correlation analysis, we excluded the three patients that did not have LCI measurements. Pearson correlation was used for the relation between IgG and the quantitative variables. The normality of distribution was checked by the Kolmogorov–Smirnov test. IgG levels were compared between groups for categorical parameters by *t*-test (for normal distribution) or Mann Whitney test (for non-normal distribution). *p* < 0.05 was considered to be statistically significant.

## 3. Results

We found a positive correlation between IgG and LCI (r = 0.342, *p* = 0.005).

Table 1 presents the patient characteristics. As can be seen, mean age was 20.5 ± 11.6 years. Pancreatic status was almost evenly distributed (approximately half the patients were PI and half PS). Seventeen patients carried the F508del mutation, and another three were homozygous for F508del. CT scans were available for 55 patients. Mean CT and SK scores were 9.5 ± 5.6 and 63.1 ± 10.6, respectively. Chronic colonization with PA was seen in 43.5%. The most prevalent co-morbidity was CF liver disease (26%), followed by CFRD (15%). The patients received a total of 20.4 ± 21.9 days of antibiotics in the year preceding the study visit. Regarding the pulmonary functions, FEV1 was slightly reduced (mean 76.9 ± 22.2% predicted) and LCI was elevated (mean 10.0 ± 2.9).

Mean IgG levels were 1372.65 ± 506.4 mg/dL, and 19 (27.5%) patients had elevated IgG. CRP values were available for 58 patients. Median CRP was 0.34 (0.11–1.43) mg/dL (normal range 0–0.5). Eleven patients had *Hemophilus influenzae* (*H. influenzae*) and 19 Staphylococcus (Staph.) in their sputum cultures. Seventeen patients received chronic therapy with azithromycin (three times a week).

In the analysis of the categorical parameters, IgG was higher in PI patients (1504.3 ± 625.5 vs. 1229 ± 276.1 in PI vs. PS, *p* = 0.023) and patients with chronic liver disease (1702.6 ± 720.3 vs. 1256.2 ± 345.5 with vs. without liver disease, *p* = 0.001). IgG levels were slightly higher in patients who received chronic azithromycin therapy (1420 ± 495.1 vs. 1235 ± 503.8, *p* = 0.046). IgG levels were similar in patients who carried at least one F508del mutation (homo/heterozygous) and those who did not carry the mutation. IgG did not correlate with chronic bacterial colonization with PA, *Hemophilus influenzae* or Staph., or the presence of CFRD. The small number of patients with gastrostomy (*n* = 5) and ABPA (*n* = 4) precluded correlation analysis.

**Figure 1:**
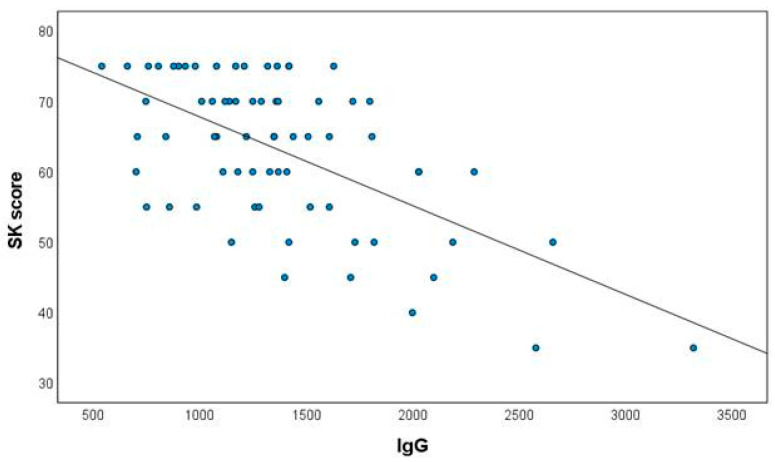
The correlation between IgG and SK score. SK score = Shwachman–Kulczycki score. r = −0.613, *p* < 0.001.

Table 2 presents the correlations between IgG and the other parameters for the 66 patients who had LCI measurements. In addition to LCI, positive correlations were also found with CRP and days on antibiotic treatment in the previous year—PO, IV and total. IgG correlated negatively with spirometry results—FVC%, FEV1% and FEF 25–75%. IgG also correlated positively with CT score and negatively with SK score. Figure 1 presents the correlation between IgG and SK score.

## 4. Discussion

In this single-center retrospective study, we evaluated the association between IgG and LCI as markers of disease activity compared with others known markers of disease severity in CF patients. We found positive correlations with LCI. IgG was elevated in approximately one quarter of our patients and was higher in patients with pancreatic insufficiency and liver disease. IgG correlated with additional multiple parameters of disease severity. We found positive correlations with CRP, as well as CT scores and number of days of antibiotic treatment, and negative correlations with spirometry results and SK score. These findings support the use of IgG as a marker of disease activity.

We found correlations between IgG and LCI, as well as spirometry, clinical parameters (including SK score), and CT scores. In concordance with our findings, IgG was found to be correlated with FEV1 in several studies [18,19]. We are not aware of studies that examined the correlation with LCI. In the study mentioned earlier, hypogammaglobulinemia was associated with better chest X-ray scores [20]. Garside et al. found lower FEV1, worse SK scores and worse chest X-ray scores in patients with elevated IgG compared to controls [21]. We are not aware of studies evaluating IgG and CT scores. Taken together, our results imply that higher IgG levels are clearly associated with a more severe disease, manifested by clinical parameters, lung function results and imaging findings.

IgG levels were elevated in 27.5% of our patients, rates similar to those reported in the literature. In the early 1980s, Matthews found hypogammaglobulinemia G in 22% of younger patients (<10 years), while 25% of older patients had hyperglobulinemia. Patients with hypogammaglobulinemia G had milder lung disease [20]. In a follow-up study, levels of IgG predicted morbidity and mortality over five years [22]. In another small study in CF adults, 61% had elevated IgG [6], while the frequency of hypergammaglobulinemia G in a pediatric cohort increased from 16% to 25% in four years [18].

Serum levels of IgG have been found to be clearly associated with the progression of lung disease in CF. The mechanism is not clear, and some studies suggested a causal role. IgG levels may reflect a hyperimmune state that is ineffective against infection and possibly destructive to the airways [19]. One of the hypothetical mechanisms is local deposition of pro-inflammatory immune complexes in the lungs [6,22]. Animal models found a mitogenic effect of PA antigens, resulting in polyclonal B-cell activation and generalized immunoglobulin secretion [6]. The inability of the immune effectors to clear PA effectively appears to result in continued B- and T-cell stimulation, which contribute to lung damage [19].

CF patients who were not infected with PA were also found to have an increased prevalence of high IgG and IgG4 [6]. In a pediatric cohort, there was no correlation between IgG and chronic PA [18]. In our cohort, almost half the patients had chronic PA in their sputum, and there was no correlation with IgG. Thus, PA infection appears to be a major, but not the only, factor causing hypergammaglobulinemia in CF

In this study, we found correlations between IgG and CRP levels. CRP is a marker of inflammation, which has been found to increase during pulmonary exacerbations and decrease after antibiotic therapy [19]. Even in stable patients, higher CRP levels were associated with worse clinical and quality of life scores at a given FEV1 [23]. Higher IgG and CRP levels were found to be correlated with disease severity and exercise capacity [4,19]. However, IgG levels were better predictors of longitudinal changes, thus may better represent a chronic inflammatory state [4].

When examining the categorical parameters, we found that PI patients and patients with liver disease had higher IgG levels. In the context of inflammation and CF liver disease, Fiorotto et al. suggested an interesting mechanism. In CFTR-defective cholangiocytes, Src tyrosine kinase activates toll-like receptor 4, resulting in activation of B cells and pro-inflammatory cytokine production in response to endotoxins [24]. However, Matthews et al. found similar liver functions tests and hemoglobin A1C levels in patients with hypo- and hypergammaglobulinemia [20].

In our study, neither CFRD nor the presence of F508del mutation correlated with IgG. We found only one study that related to the presence of F508del. IgG and CRP correlated with FEV1, and results were unchanged by adjusting for the number of F508del alleles [19].

The strengths of this study include assessment of correlation between IgG and multiple parameters of disease severity. Some correlations, such as LCI and CT score, were not previously reported. The main limitation of our study is the relatively small number of patients. Some of the co-morbidities (such as gastrostomy and ABPA) were present only in a few patients, precluding sub-group analysis. The study was retrospective, and not all evaluations were performed at a single visit. However, the evaluations were within an acceptable time range. The design was cross-sectional; thus, we could not assess changes in IgG levels over time. We did not have the subtypes of IgG in our data, therefore could not evaluate correlation between subtypes of IgG and disease severity. Levels of other inflammatory markers, such as cytokines, were not available. The correlation coefficient (r) was low in the analyses: thus, correlations may be statistically significant, but the scatter may be too wide to be of clinical significance.

## 5. Conclusions

In conclusion, we found that IgG correlated positively with LCI and several other important markers of disease severity. However, IgG determination is still an auxiliary test, complementing other tests, such as lung function (including LCI) and imaging tests. Further prospective longitudinal studies are needed, with a larger number of patients, assessing IgG levels at baseline, during exacerbations and in patients receiving mutation –specific therapies.

## Figures and Tables

**Table 1:** Patient characteristics.

	CF (*n* = 69)
Age (years)	20.5 ± 11.6
Male (%)	39 (56.5%)
PI/PS	36/33
BMI (kg/m^2^)	20.8 ± 4.9
CT score (*n* = 55)	9.5 ± 5.6
SK score	63.12 ± 10.6
CFRD	10 (15%)
CF liver disease	18 (26%)
Gastrostomy	5 (7%)
ABPA	4 (6%)
Chronic PA	30 (43.5%)
Days of oral/IV AB	14.6 ± 17.0/5.8 ± 9.8
FVC (L; % pred)	2.9 ± 1.2; 83.0 ± 19.0
FEV1 (L; % pred)	2.36 ± 1.08; 76.9 ± 22.2
FEF 25-75 (L; % pred)	2.2 ± 1.4; 65.3 ± 35.5
LCI (*n* = 66)	10.0 ± 2.9
IgG (mg/dL)	1372.7 ± 506.4
CRP * (mg/dL)	0.34 (0.11–1.43)

Notes: * Median (IQR). Values are presented as mean ± SD. PI = pancreatic insufficient; PS = pancreatic sufficient; BMI = body mass index; CT = computed tomography; SK score = Shwachman–Kulczycki score; CFRD = Cystic fibrosis related diabetes; ABPA = allergic bronchopulmonary aspergillosis; PA = *Pseudomonas aeruginosa*: AB = Antibiotics; IV = intravenous; FVC = forced vital capacity; FEV1 = forced expiratory volume in one second; pred = predicted; FEF25-75 = forced expiratory flow between 25% and 75% of FVC; LCI = lung clearance index; CRP = C-Reactive Protein; IQR = interquartile range.

**Table 2:** Correlations of IgG with markers of disease severity.

	r	*p* Value
FVC (%)	−0.506	<0.001
FEV1 (%)	−0.527	<0.001
FEF25–75 (%)	−0.503	<0.001
LCI	0.342	0.005
CT score (*n* = 55)	0.435	0.001
SK score	−0.613	<0.001
Days of oral AB	0.244	0.049
Days of IV AB	0.321	0.009
CRP (*n* = 58)	0.38	0.003

FVC = forced vital capacity; FEV1 = forced expiratory volume in one second; FEF25–75 = forced expiratory flow between 25% and 75% of FVC; LCI = lung clearance index; CT = computes tomography; SK score = Shwachman–Kulczycki score; AB = Antibiotics; IV = intravenous; CRP = C-Reactive Protein.

## Data Availability

The data presented in this study are available on request from the corresponding author.
